# C-reactive protein, Epstein-Barr virus, and cortisol trajectories in refugee and non-refugee youth: Links with stress, mental health, and cognitive function during a randomized controlled trial

**DOI:** 10.1016/j.bbi.2019.02.015

**Published:** 2020-07

**Authors:** Catherine Panter-Brick, Kyle Wiley, Amelia Sancilio, Rana Dajani, Kristin Hadfield

**Affiliations:** a Department of Anthropology, Yale University, New Haven, USA; b Department of Biology and Biotechnology, Hashemite University, Zarqa, Jordan; c Department of Biological and Experimental Psychology, Queen Mary University of London, UK

**Keywords:** Inflammation, Cortisol, Immune function, Refugee, War, Mental health, Stress, Randomized control trial, Adolescent, Life history

## Abstract

Experiencing childhood adversity has been associated with significant changes in inflammation, cell-mediated immunocompetence, and cortisol secretion. Relatively few studies have examined, longitudinally, alterations to inflammatory processes during adolescence, especially outside Western contexts; none have evaluated biomarker trajectories for at-risk youth in response to a structured behavioral intervention. We conducted a randomized controlled trial evaluating the efficacy of a humanitarian intervention targeting stress-alleviation, with 12–18 year-old Syrian refugees (*n* = 446) and Jordanian non-refugees (*n* = 371) living side-by-side in war-affected communities in Jordan. We measured C-reactive protein (CRP), Epstein-Barr virus antibodies (EBV), and hair cortisol concentration (HCC) at three timepoints (pre/post intervention and 11 month follow-up), and assessed three main outcomes (psychosocial stress, mental health, and cognitive function). Using growth mixture models, regressions, and growth curve models, we identified three distinct trajectories for CRP, two for EBV, and three for HCC, and examined their associations with age, gender, BMI, poverty, and trauma. We found associations with BMI for CRP, refugee status for EBV, and BMI and gender with HCC trajectory. In terms of health outcomes, we found associations between rising CRP levels and perceived stress (*B* =  −2.92, *p* = .007), and between HCC hypersecretion and insecurity (*B* = 7.21, *p* = .017). In terms of responses to the intervention, we observed no differential impacts by CRP or EBV trajectories, unlike HCC. These results suggest that commonly-assayed biomarkers do not associate with health outcomes and respond to targeted interventions in straightforward ways. Our study is the first to examine multiple biomarker trajectories in war-affected adolescents, in order to better evaluate the extent, timing, and malleability of the biological signatures of poverty, conflict, and forced displacement.

## Highlights

• We observed multiple CRP, EBV, and HCC trajectories, indicating within-population heterogeneity.
• Refugees and non-refugee adolescents showed similar CRP and HCC trajectories.
• Rising CRP levels were related to perceived stress, high HCC to insecurity.
• Post-intervention, HCC reduced by more than one third, while CRP and EBV did not.

## 1 Introduction

Experiencing childhood adversity has been linked to changes in inflammation, cell-mediated immunocompetence, and cortisol secretion, and robustly associated with poor health outcomes over the lifecourse (Danese and McEwen, 2012). Relatively few studies have examined, longitudinally, these biological profiles during adolescence, which would help clarify the mechanisms that generate health vulnerability (Slopen et al., 2013b). Extant studies include the U.S. National Longitudinal Study of Adolescent to Adult Health (ADD Health) (Harris et al., 2009), the U.K. Avon Longitudinal Study of Parents and Children (ALSPAC) (Boyd et al., 2013), and the Dutch Tracking Adolescents’ Individual Lives’ Survey (TRAILS) (Huisman et al., 2008). Notably, there is limited biobehavioral research conducted outside Western contexts, work that would help elucidate the short- and long-term significance of a broader range of adverse life experiences (Kohrt et al., 2015, McDade, 2002, Worthman and Panter-Brick, 2008). This empirical work will inform our understanding of the functional implications of potential tradeoffs, a central concept in life history theory and evolutionary biology (Del Giudice and Gangestad, 2018). To our knowledge, no research with at-risk adolescents has yet assessed which biomarkers best track changes in wellbeing and cognitive function in response to a discrete behavioral intervention. In this paper, we evaluate multiple biomarkers with respect to their associations with psychosocial stress, mental health, and cognitive function, in response to a brief, structured intervention targeting levels of ‘toxic stress’ for adolescents living in environments of extreme adversity. We focus on measuring three pathways – inflammation, cell-mediated immune function, and neuroendocrine stress – through which adverse experiences ‘get under the skin.’

Recent reviews have shown that biomarkers are not always straightforward indicators of childhood adversity, health vulnerability, and biological adaptability. With regard to inflammatory responses, research to-date offers mixed results (Slopen et al., 2012). While C-reactive protein (CRP) is a well-established measure of systemic inflammation, it is likely that CRP reactions will vary in response to different types of adversity, and across the period of adolescence. In a cohort of British children, for example, Danese et al. (2011) found associations between childhood maltreatment and CRP levels at age 12, conditional on the development of depression, while Baldwin et al. (2018) associated victimization with CRP for girls, but not boys, at age 18. Other studies have documented null associations between socio-economic status and CRP levels for 10–11 year-old British school-children (Cook et al., 2000), Finnish children and adolescents (Gimeno et al., 2008), and Canadian 15–29 year olds (Miller et al., 2009), or even inverse associations in British adolescents (Thomas et al., 2005). Links between CRP and posttraumatic stress disorder (PTSD) or cognitive function also remain unclear. For example, in a cross-sectional study of adult Iraqi refugees, Sondergaard et al. (2004) found lower CRP levels in refugees with PTSD, whereas Canetti et al. (2014) observed elevated CRP in Israeli citizens who developed PTSD in the wake of rocket attacks. And while longitudinal studies of young adults by Jonker et al., 2014, Cohen-Manheim et al., 2015 found null associations between CRP and cognitive function, the study of British 11–14 year-olds by Cullen et al. (2017) found that CRP predicted poor memory and executive function, even after adjusting for emerging psychopathology. Del Giudice and Gangestad (2018) have urged a re-evaluation of the role of inflammatory response given that “patterns of immune activity that have been regarded as pathological or dysregulated may represent adapative […] responses to environmental threats” (p.71). The call here is to better understand patterns of human biological variation and their associations with health outcomes over the life course, which includes a careful empirical examination of biological profiles for low- and high-risk youth during adolescence.

Cell-mediated immunocompetence has also demonstrated a complex association with social experiences and measures of health vulnerability. A body of work has focused on antibodies of the Epstein-Barr virus (EBV), infecting approximately 90% of the world’s population (Cohen, 2000) as markers of immunocompetence. EBV titers are “one of the strongest immune-related correlates of psychosocial stress” (Slopen et al., 2013b, p. 64): they rise with acute or chronic stressors, due to a suppression of immune function. One of the first cross-cultural studies of EBV was conducted in Western Samoa (McDade, 2002) during a period of rapid sociocultural and economic change: for young people (10–20 year olds), status incongruity was associated with increased EBV levels, indicating compromised immune function. In Afghanistan, EBV levels were markedly different by gender; for women, they mapped onto the intensity of family-level stressors, reflecting the strict control of their public behavior (Panter-Brick et al. 2008). These studies show that EBV levels can help track responses to frustrating social experiences and challenges to social status. People with different exposures to social adversity, especially social inequality, show different patterns of immune function, and by extension, susceptibility to ill-health (Kiecolt-Glaser and Glaser, 1981).

Neuroendocrine stress responses vary considerably in their associations with lifetime adverse experiences, leading to multiple patterns of dysregulation of the hypothalamic–pituitaryadrenal (HPA) axis. In studies of chronic stress, hair cortisol concentrations (HCC) have emerged as an important non-invasive marker of HPA axis activity (Russell et al., 2012, Stalder et al., 2017), and given that HCC from the 1 cm of hair closest to the scalp reflect HPA activation over the previous month, taking repeated samples for an individual over time is thus akin to appraising a physiological stress diary. However, the links between cortisol levels and self-reported data on adverse experiences are far from straightforward, given that there may be several different patterns of cortisol responses and/or dysregulation in response to chronic stress, which likely affect overall concentrations measured in the body (Boyce and Ellis, 2005, Staufenbiel et al., 2013, Steudte-Schmiedgen et al., 2016). Thus traumatic experiences and chronic stress can result in either elevated or blunted cortisol secretion (Steudte-Schmiedgen et al., 2016). For example, elevated HCC has been consistently associated with war-related stress, particularly for subgroups with PTSD, including adolescents growing up in Palestine (Shaheen et al., 2018), young adults living in camps for internally displaced people in northern Uganda (Steudte et al., 2011), female university students in Libya (Etwel et al., 2014), and adult asylum seekers in Germany (Mewes et al., 2017). Conversely, inverse associations between HCC and traumatic experiences have been documented in studies examining lifetime trauma and adverse childhood experiences (Hinkelmann et al., 2013, Steudte et al., 2013, Kalmakis et al., 2015). Both patterns of dysregulation raise important long-term health concerns: chronically high cortisol levels are associated with mental health, memory and learning deficits, and metabolic regulation, while chronically low cortisol levels compromise immune function (Russell et al., 2012, Staufenbiel et al., 2013, Greff et al., 2018).

Using data from a randomized controlled trial, we evaluated the usefulness of multiple biomarkers as indicators of adverse experiences, health vulnerability, and biological adaptability in response to an intervention. Our cohorts were 12–18-year-old Syrian refugees and Jordanian non-refugees, living side-by-side in urban centers close to the border. There are compelling reasons for examining markers of inflammation, immunocompetence, and hormone regulation in war-affected youth. In the wake of the Syria and Iraq wars, studies have highlighted the burden of loss, trauma, and toxic stress in child and adolescent refugees, which places a whole generation at risk of poor health and worsened social and developmental outcomes (Save the Children, 2018). Examination of biomarkers in young refugees is scant, despite their potential utility and the global importance of this population (people under 18 make up 52% of refugees worldwide; UNHCR, 2018). Now in its seventh year, the Syrian crisis has forced over 5.6 million people (45.0% under 18) to leave Syria, while a further 6.5 million Syrians (43.1% under 18) are internally displaced; over 671,000 Syrians have taken refuge in Jordan (UNHCR, 2019).

**Fig. 1:**
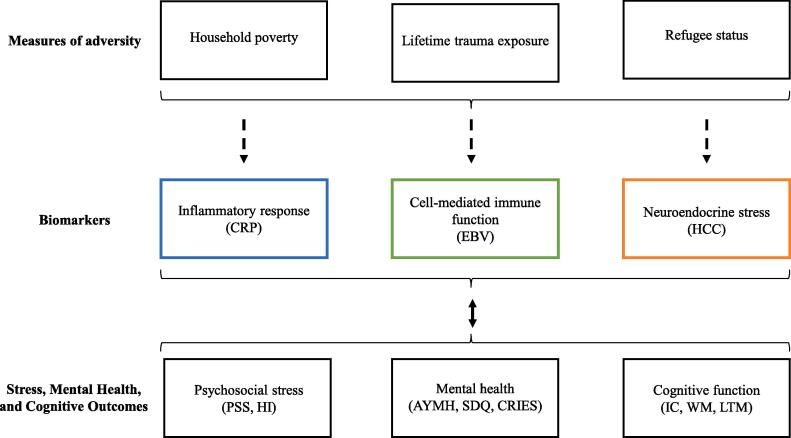
Associations between adversity, biomarkers, and outcomes*. Note:* CRP = C-reactive protein, EBV = Epstein-Barr virus, HCC = hair cortisol concentration, AYMH = Arab Youth Mental Health scale, SDQ = Strengths and Difficulties Questionnaire, CRIES = Child Revised Impact of Events Scale, PSS = perceived stress scale, HI = Human Insecurity scale, IC = inhibitory control, WM = working memory, LTM = long-term memory.

We examined associations between markers of inflammatiory response, cell-mediated immunocompetence, and neuroendocrine stress (the CRP, EBV, HCC biomarkers), with three types of outcomes (self-reported psychosocial stress, self-reported mental health, and tests of cognitive function) over three timepoints. Our observations spanned nearly a year: pre-intervention, 12 weeks after baseline (post-intervention), and 11 months after baseline (Supplemental Figure A). In this paper, we present new data on CRP and EBV and draw on previously published work documenting intervention impacts on HCC, self-reported symptoms of psychosocial stress and mental health, and tests of cognitive skills (Dajani et al., 2018, Panter-Brick et al., 2018). We examined three main research questions. *First (RQ1), what are the trajectories of inflammation, immunocompetence, and cortisol secretion in this cohort?* Given the relative dearth of longitudinal work in war-affected adolescents, we did not have *a priori* hypotheses on the nature or number of trajectories. We examined associations of these trajectories with measures of adversity (household poverty, trauma exposure, refugee status) and demographic covariates (age, gender, BMI). *Second (RQ2), what are the associations between changes in biomarkers and psychosocial stress, mental health, and cognitive function*? We hypothesized that changes in the levels of biomarkers would mirror trends towards improved or worsened outcomes across the period of study. Our analysis was conceptually guided by the expected associations illustrated in Fig. 1. *Third (RQ3), which biomarkers show specific malleability over the short-term, in response to the intervention?* We hypothesized that participants who engaged in the intervention would show reduced CRP, EBV, and HCC, indexing a beneficial regulation of inflammatory processes, immune competence, and neuroendocrine stress. Such research helps to build knowledge on biological profiles during adolescence and their associations with life adversity and health outcomes.

## 2 Methods

### 2.1 Study design

We evaluated the *Advancing Adolescents* (Arabic: *Nubader*) program, a community-based intervention implemented by Mercy Corps for youth affected by the Syria and Iraq crises in Jordan, Lebanon, Iraq, Syria, and Turkey (Mercy Corps, 2016) as part of the *No Lost Generation* initiative. The program is structured to provide safety, support, and group-based activities, targeting both refugee and non-refugee youth. It explicitly draws on neuroscience to communicate an understanding of the ‘emotional brain’ in response to experiences of profound stress, in order to help youth manage impulses, assess risk, and approach the future (MacPhail et al., 2017). A wait-listed randomized control trial (ClinicalTrials.gov ID: NCT03012451) was conducted to evaluate program impacts; in the first wave (*n* = 214), participants were quasi-randomized based on their ability to participate before the start of Ramadan, a month which marked the end of one cycle of humanitarian funding; in the second wave (*n* = 603), just after Ramadan, participants were fully randomized through a coin toss allocation (ratio 1:1). Intervention and evaluation were conducted by separate teams, although fieldworkers were not blind to group assignment. Participants (*n* = 446 refugees, *n* = 371 non-refugees) were 12 to 18 years old (*M* = 14.49, *SD* = 1.79) and 56.9% male. Youth in the treatment (56.7%) and control groups were strictly comparable.

Data were collected immediately before the start of the intervention (T1), just after the 8-week intervention (T2), and at 11.3 months to capture a longer-term follow-up (T3). We collected socio-demographic data at baseline, biological samples and psychosocial outcomes at all three timepoints, and tested cognitive function pre- and post-intervention (Supplemental Figure A); participants completed all measures sequentially on the same day. Sampling levels were powered to detect small-to-moderate intervention effect sizes (*d* = 0.3). Eight Syrian and Jordanian fieldworkers were responsible for biomarker samples, face-to-face interviews, and cognitive function testing, with 4 supervisors checking data and storing samples on a daily basis. All survey measures were previously used with war-affected adolescents. This study received formal approval from the Prime Minister’s Office of Jordan and ethics approval from [blinded] University.

#### 2.1.1 Measures

##### 2.1.1.1 Measures of adversity

Trauma exposure, household poverty, and refugee status were three main measures of adversity. All were assessed through participant self-report. Lifetime trauma exposure was measured with the 21-item Trauma Events Checklist, which was adapted for use with war-exposed youth in the Middle East (Panter-Brick et al., 2009). This measure is a yes/no checklist of lifetime exposure to trauma events (such as exposure to bombardments/rocket explosions; seeing someone else beaten, shot, or killed; or having their home forcibly searched by the police or an armed militia). Relative poverty was measured with a 12-item Household Wealth Index of material goods, designed to capture local wealth differentials in Jordan. In this paper, we use the term refugee status, rather than the term nationality, to distinguish between Syrian refugees and Jordanian non-refugees.

##### 2.1.1.2 Participant characterictics

BMI, age, and gender were the main covariates. Height and weight data were measured using standard field procedures, to determine BMI (kg/m^2^). Due to challenging field conditions (no electricity or cool air flow in hot summer months), and participant fatigue, we made the anthropometry optional (resulting in BMI data on 452 participants), and opted not to collect morbidity data, to avoid undue burden on participants.

##### 2.1.1.3 Biomarkers

We used blood spots to collect CRP and EBV data; we followed standard protocols (McDade et al., 2007) to collect 2–5 drops of blood on standardized filter paper. Fingers were cleaned with alcohol wipes and pricked with sterile disposable lancets. Blood spot samples were dried at room temperature, transported the same day for storage at −20 °C in Amman, then shipped for storage at −80 °C to the USA for analysis at the Yale Reproductive Ecology Laboratory (YREL), at Yale University. Hair samples were collected by professional hairdressers, who cut ∼100 strands on the vertex posterior scalp to determine HCC, and sent to Canada for analysis at the Drug Safety Laboratory (DSLab) of the Robarts Research Institute at the University of Western Ontario. Samples were shipped to the laboratory in regular batches after collection.

We quantified CRP and EBV using previously published EIA and ELISA protocols validated for use with dried blood spots (Eick et al., 2016, McDade et al., 2004). Pre- and post-intervention samples were run on the same plate, while 1-year follow-up samples were run as one batch. We assayed HCC using hair segments (0–2 cm at baseline, 0–1 cm at T2, 0–1 cm at T3), weighed and minced before undergoing ELISA testing using a modified, commercially available ALPCO Diagnostics salivary cortisol test kit (11-CORHU-E01-SLV) (Dajani et al., 2018, Panter-Brick et al., 2018, Greff et al., 2018). For HCC, samples from different timepoints were run on separate plates.

EIA and ELISA analyses use standards that are run on each plate for the generation of a plate-specific standard curve; individual sample concentrations are calculated from each curve. Samples were run in duplicate and those with intra-assay coefficients of variation greater than 10% were re-analyzed. Hair cortisol samples falling outside the range of the plate-specific standard curve were re-analyzed, extracting a greater mass of hair or diluting the extracted sample to bring the sample into the detectable range. The lowest limits of assay detection were 0.05 mg/L for CRP and 22.06 Au/mL for EBV. A lower limit of detection could not be calculated for HCC as the assay results are dependent on the variable mass of hair used. The inter-assay coefficients of variation across plates for CRP, EBV, and HCC were 15.6%, 17.1%, and 8.6% respectively. Using a chi-square analysis, we tested for batch effects using the distribution of hyposecretion cortisol results and found none (*p* > 0.05). Assays indicated good sensitivity, precision, and reliability.

#### 2.1.2 Outcomes: psychosocial stress, mental health, and cognitive function

Surveys were individually completed with the help of fieldworkers in dedicated, private spaces in community centers; fieldworkers read questions aloud to participants, in Arabic, and noted their responses. Levels of psychosocial stress were measured with the 14-item Perceived Stress Scale (PSS, Almadi et al., 2012) and the 10-item Human Insecurity scale (HI, Ziadni et al., 2011). PSS is a measure of past-month feelings of being upset and unable to cope; while developed in Western contexts (Cohen et al., 1983), it has been validated in Jordan (Almadi et al., 2012). HI is a measure developed and validated in the West Bank to ascertain insecurity pertaining to safety and access to daily life necessities.

We assessed mental health problems with the 21-item Arab Youth Mental Health scale (AYMH, Mahfoud et al., 2011), the 20-item Total Difficulties subscale of the Strengths and Difficulties Questionnaire (SDQ, Goodman and Goodman, 2009), and the 8-item Child Revised Impact of Events scale (CRIES, Punamaki et al., 2015, Veronese and Pepe, 2013). AYMH is a regionally-developed measure of anxiety/depression symptoms, while SDQ is an internationally-developed but locally validated (Almaqrami and Shuwail, 2004, Thabet et al., 2000) measure of emotional and behavioral difficulties. CRIES measures the severity of post-traumatic stress symptoms.

Cognitive function was measured via the RACER set of short cognitive tests, designed for use in low- and middle-income countries on tablet computers (Hamoudi & Sheridan, 2015). These game-like tasks measure inhibitory control (IC, the ability to suppress a dominant response in order to carry out a non-dominant response), working memory (WM, the ability to hold, update, and manipulate information for a short period of time), and long-term memory (LTM, the ability to hold information in the mind for a long period of time). Cognitive function data were collected immediately following completion of the demographic and psychometric questionnaires; this was overseen by the same fieldworker who had conducted their interview. All fieldworkers received extensive training in the use of RACER tasks.

#### 2.1.3 Statistical analyses

We removed CRP values ≥ 10 mg/L to avoid including participants with active infections (as in Pearson et al., 2003): *n* = 22 (2.9%) at T1, *n* = 6 (1.3%) at T2, and *n* = 7 (2.9%) at T3. In line with other work (e.g. Skoluda et al., 2012), we excluded cortisol values more than + 2SD from the mean: *n* = 5 outliers at T1, *n* = 15 at T2, and *n* = 26 at T3 (pre-log HCC exceeding 90 pg/mg). There were no differences in data exclusion by gender or refugee status. To normalize distributions, we log-transformed CRP, EBV, and HCC.

We used latent growth mixture modeling (MPlus v.7.3) to test for multiple CRP and EBV trajectories in our cohort (RQ1). We used HCC trajectories established in previous work (Dajani et al., 2018). To determine the optimal number of trajectories, we added them one at a time and tested for improved model fit. Slopes and intercepts were allowed to vary freely across trajectories, but were held constant within groups. We used Ram and Grimm’s (2009) technique for selecting the optimal number of latent classes within our sample over time, first examining Akaike Information Criteria (AIC) and sample-size adjusted Bayesian Information Criteria (SSA-BIC), then Vuong-Lo-Mendell-Rubin (VLMR) likelihood ratio tests and Adjusted Lo-Mendell-Rubin (aLMR) tests to assess whether the model fit better than the model with k-1 trajectories. Our analyses identified three trajectories of CRP and HCC, and two trajectories of EBV (Table 2). For CRP, the four-class model did not have significant VLMR or aLMR values, so we did not test models with additional classes; similarly, for EBV, the three-class model did not have significant VLMR or aLMR values and so we did not continue further (Jung and Wickrama, 2008).

We then tested for associations, over time, between biomarker trajectories relative to health outcomes (RQ2). We used growth curve models to see how psychosocial stress (PSS, HI) and mental health (AYMH, SDQ, CRIES) outcomes differed at intercept and over time, as a function of biomarker trajectories (RQ2). We controlled for measures of adversity and demographic confounders; inclusion of control variables was informed both by previous research and analyses of our dataset. To identify predictors of biomarker trajectories, we ran separate logistic regressions with the trajectories of our three biomarkers (CRP, EBV, HCC) as the outcome variables; these regressions included measures of adversity (poverty, trauma exposure, and refugee status) and other potential covariates (BMI, age, and gender) as predictors. Household poverty and trauma were not related to CRP, EBV, or HCC trajectory. As expected, BMI was associated with CRP and HCC, but not EBV. We also found that gender was related to HCC and refugee status to EBV. Subsequent analyses (RQ2 and RQ3) included the significant measures of adversity and covariates. We used regressions for cognitive outcomes (IC, WM, LTM), to accommodate data collected at T1 and T2 (not T3): we tested differences in cognitive function at T1 by CRP or HCC trajectories, and then whether their trajectories predicted change from T1 to T2. Controlling for BMI, we assessed whether CRP trajectories were predictive of psychosocial stress, mental health problems, or cognitive function, in line with predicted relationships in Fig. 1. Controlling for refugee status, we assessed whether EBV predicted psychosocial stress. Controlling for gender and BMI, we assessed whether HCC trajectories were predictive of psychosocial stress, mental health problems, or cognitive outcomes.

Finally, we tested for longitudinal effects of the intervention on biomarkers (RQ3). To assess whether the intervention impacted inflammation, immune response, or neuroendocrine stress (RQ3), we ran growth curve models with participation in the intervention as a time-varying predictor variable. Finally, we tested whether intervention effects on CRP, EBV, or HCC were moderated by either trauma exposure or gender.

## 3 Results

### 3.1 Sample characteristics

Participants were 817 Syrian refugee and Jordanian non-refugees (age 12–18) living in Northern Jordan. Descriptive statistics are presented in Table 1 and Appendix Table A. There was no difference between Syrians and Jordanians in terms of age (14.33 vs. 14.43 years), gender (55.4% vs. 58.8% male), or BMI (21.07 vs. 21.34 kg/m^2^) at T1. As expected, Syrian refugees reported more lifetime trauma exposure (6.36 vs. 1.08 events), greater poverty (6.27 vs. 10.00 household items), perceived stress (28.73 vs. 25.94 PSS symptom scores), insecurity (67.87 vs. 60.28 HI scores), anxiety and depression (35.90 vs. 31.91 AYMH scores), emotional and behavioural difficulties (15.55 vs. 14.04 SDQ scores), and symptoms of posttraumatic stress (19.59 vs. 5.42 CRIES, all *p* < .001). There were no differences between Syrians and Jordanians in cognitive skills, measured by tests of inhibitory control, working memory, or long-term memory.

**Table 1:** Participant characteristics at baseline (T1).

	Syrian refugee				Jordanian non-refugee				Combined sample			
	Mean	*SD*	Median	*n*	Mean	*SD*	Median	*n*	Mean	*SD*	Median	*n*
*Demographics*												
Age (years)	14.33	1.82	14.00	446	14.43	1.59	14.00	371	14.37	1.72	14.00	817
BMI (kg/m^2^)	21.07	4.16	20.42	262	21.34	4.67	20.42	190	21.19	4.38	20.42	452
Lifetime trauma exposure (*n* events)	6.36	3.25	6.00	446	1.08	1.63	0.00	371	3.96	3.73	3.00	817
Socioeconomic status (household items)	6.27	2.24	6.00	404	10.00	2.05	10.50	336	7.96	2.85	8.00	740

*Stress and mental health outcomes*												
Perceived stress scale (PSS)	28.73	5.88	28.50	444	25.94	6.31	26.00	371	27.46	6.23	27.00	815
Human insecurity (HI)	67.87	19.72	70.00	444	60.28	22.37	60.00	371	64.41	21.29	66.67	815
Arab Youth Mental Health (AYMH)	35.90	8.66	35.00	446	31.91	7.92	30.00	370	34.09	8.56	32.00	816
Strength and Difficulties (SDQ)	15.55	6.06	15.00	446	14.04	6.22	14.00	370	14.87	6.17	15.00	816
Posttraumatic stress (CRIES)	19.59	10.99	20.00	444	5.42	9.95	0.00	371	13.14	12.67	12.00	815

*Cognitive outcomes (challenge trials)*												
Inhibitory control (IC)	0.84	0.28	0.97	203	0.86	0.25	0.97	184	0.85	0.27	0.97	387
Working memory (WC)	67.94	28.98	63.86	203	67.74	24.04	67.77	184	68.80	26.73	65.35	387
Long-term memory (LTM)	0.42	0.19	0.42	203	0.43	0.18	0.42	184	0.43	0.18	0.42	387

*Biomarkers*												
C-reactive protein (CRP), mg/L	1.30	1.78	0.56	399	1.56	1.90	0.70	328	1.42	1.83	0.62	727
Epstein-Bar virus antibody (EBV), U/ml	144.07	142.04	90.42	309	170.82	155.09	102.51	269	156.94	148.79	97.11	578
Hair cortisol concentration (HCC), pg/mg	10.04	10.95	6.70	352	8.80	8.47	6.30	259	9.52	9.98	6.50	611

*Note*: We present raw biomarker values (minus outliers) in this table, to enable comparison with other studies; analyses use log values. Data are pre-intervention (T1), except for BMI which was measured at T1 and T3. There is a strong correlation between BMI at T1 and T3 for participants who have data at both timepoints (*r* = 0.66*, p* < .001).

As expected, biomarker values were correlated with themselves over time: moderately so, for CRP (T1-T2, *r* = 0.41, *p* < .001; T1-T3, *r* = 0.44, *p* < .001; T2-T3. *r* = 0.53, *p* < .001); strongly for EBV (T1-T2 *r* = 0.83, *p* < .001; T1-T3, *r* = 0.82, *p* < .001; T2-T3 *r* = 0.80, *p* < .001); and weakly for HCC (T1-T2, *r* = 0.26, *p* < .001; T1-T3, *r* = 0.24, *p* = .002; T2-T3, *r* = 0.19, *p* = .010). Biomarkers were not, however, correlated with each other: at T1, CRP was unrelated to either EBV (*r* = 0.08, *p* = .059) or HCC (*r* = 0.03, *p* = .422), and EBV and HCC were also uncorrelated (*r* = 0.03, *p* = .524).

### 3.2 Inflammation, immunocompetence, and cortisol trajectories

Our first set of analyses examined the trajectories of inflammation, immunocompetence, and cortisol secretion present in this cohort (RQ1). We tested for distinct trajectories with latent growth mixture modeling (Table 2) and examined their associations with participant characteristics and measures of adversity. As expected, BMI was associated with CRP and HCC. Gender was related only to HCC, while age had no impact on CRP, EBV, or HCC. While refugee status was associated with EBV, household poverty and trauma exposure were not significantly related with any biomarker trajectory. We present results for each biomarker in turn.

**Table 2:** Model fit statistics for latent class mixture models of CRP, EBV, and HCC production.

	AIC	SSA-BIC	Entropy	aLMR	VLMR
*CRP*					
2 class	1372.47	1385.73	0.71	121.68*	16.65*
**3 class**	**1345.87**	**1363.56**	**0.66**	31.04*	1.79*
4 class	1339.17	1361.27	0.64	12.10	28.49

*EBV*					
**2 class**	**267.86**	**278.95**	**0.73**	65.148*	4.72*
3 class	244.343	259.13	0.79	28.054	7.29

*HCC*					
2 class	1691.90	1704.70	0.92	29.98^**^	0.28^**^
**3 class**	**1669.25**	**1686.32**	**0.78**	27.27^**^	3.32^**^
4 class	1661.90	1683.23	0.52	12.71	11.69

*Note*: Significant aLMR and VLMR scores suggest better model fit than a k-1 class model. Based on fit indices, the 2-class model was chosen for EBV and the 3-class model was chosen for CRP. CRP = c-reactive protein, EBV = Epstein-Barr virus antibodies, HCC = hair cortisol concentration, aLMR = adjusted Lo-Mendell-Rubin likelihood ratio test, VLMR = Vuong-Lo-Mendell-Rubin likelihood ratio test. The best fitting model for each outcome is bolded.

*p < .05, ^**^p < .001

**Fig. 2:**
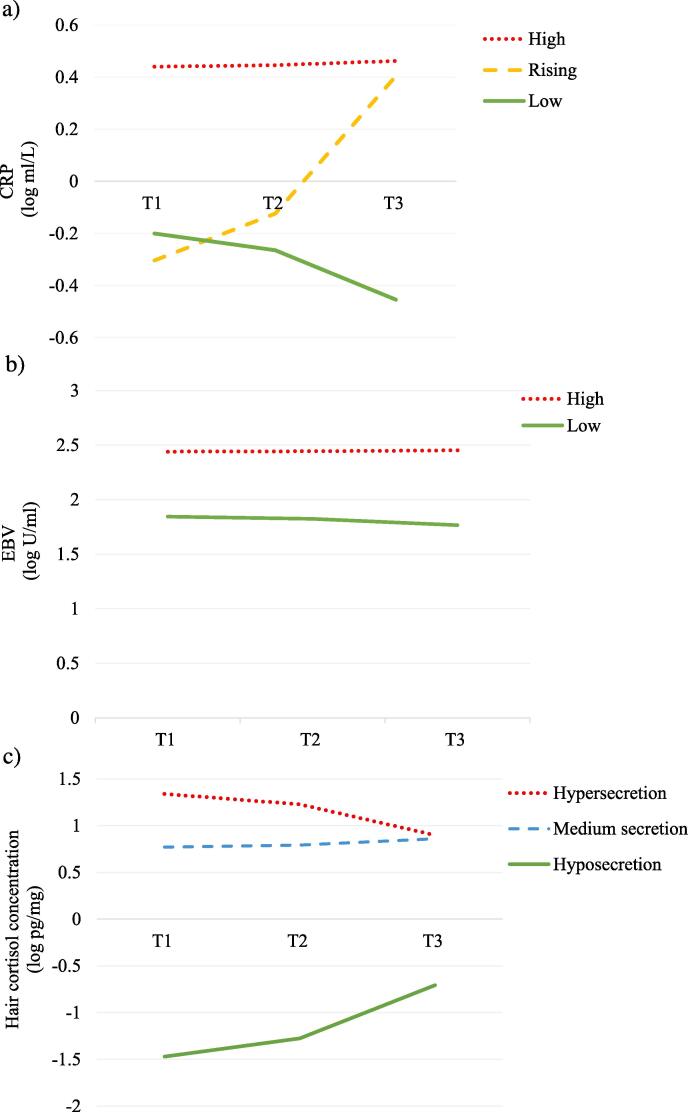
Trajectories of inflammation (a), immunocompetence (b), and neuroendocrine stress (c) in Syrian refugee and Jordanian host-community adolescents across the 11-month study period.

There were three CRP trajectories, which we labelled High, Rising, and Low (Fig. 2a). The first started relatively high and remained consistently so (*n* = 136, initial CRP = 0.440 mg/L, change per month = 0.002). The second started relatively low, and increased rapidly (*n* = 23, initial CRP = −0.304 mg/L, change per month = 0.064). The third was the largest group; it started low and had shallow decreases over time (*n* = 613, initial CRP = −0.200 mg/L, change per month = −0.023). CRP trajectory was predicted by BMI: adolescents with higher BMI were more likely to have a High CRP trajectory than a Low CRP trajectory (*B* = 0.26, *SE* = 1.06, *e^B^* = 1.29, Wald Z = 47.86, 95% CI: 1.20, 1.39, *p* < .001).

There were two EBV trajectories, which we labelled High and Low (Fig. 2b): one with sustained high values (*n* = 186), the other with lower values and shallow decreases over time (*n* = 420). In the former, initial EBV values were 2.441 U/mL and increased at a rate of 0.001 per month; in the latter, initial EBV values were 1.844 U/mL and decreased at a rate of 0.007 per month. We found differences by refugee status, with Syrians less likely to belong in the High EBV trajectory (*B* = 1.39, *SE* = 0.50, *e^B^* = 4.02, Wald Z = 7.78, 95% CI: 0.46, 2.52, *p* = .005).

There were three HCC trajectories, which we labelled Hypersecretion, Medium Secretion, and Hyposecretion (Fig. 2c). Hypersecretion started high and had shallow decreases (*n* = 70, initial cortisol = 1.340 log pg/mg, change each month = −0.040). The most common HCC trajectory, Medium Secretion, remained steady over time (*n* = 636, initial cortisol = 0.771 log pg/mg, change each month = 0.008). Hyposecretion started quite low and went up over time (*n* = 21, initial cortisol = −1.470 log pg/mg, change each month = 0.069). We found that both BMI and gender were predictors of cortisol trajectory: girls (*B* = −1.22, *SE* = 0.43, *e^B^* = 0.30, Wald Z = 8.19, 95% CI: 0.13, 68, *p* = .004) and youth with higher BMI (*B* = 0.08, *SE* = 0.04, *e^B^* = 1.08, Wald Z = 4.71, 95% CI: 1.01, 1.16, *p* = .005) were more likely to have a trajectory of HCC Hypersecretion than Medium Secretion.

We then tested whether participants with a specific trajectory in one biomarker were likely to have a similar trajectory in a different biomarker. In chi-square analyses, CRP trajectory was unrelated to either HCC (χ^2^ [4, *N* = 689] = 0.61, *p* = .962) or EBV (χ^2^ [2, *N* = 594] = 0.11, *p* = .948) trajectory. That is, having a Rising CRP trajectory, for instance, was not associated with the likelihood of having either a High or Low EBV trajectory, or any of the HCC trajectories. Similarly, HCC trajectory was not related to EBV trajectory (χ^2^ [2, *N* = 539] = 1.54, *p* = .464). Thus, participants in this cohort did not show a consistent pattern of biomarker production.

### 3.3 Associations with stress, mental health, and cognition

Our second set of analyses examined the relationships between trajectories of inflammation, immunocompetence, and cortisol secretion with outcomes of interest (psychosocial stress, mental health, and cognitive function), in order to understand how biomarkers track improved or worsened outcomes over time (RQ2). For measures of psychosocial stress, we found two main results, described in more detail below: (1) participants with different CRP trajectories had different levels of perceived stress (PSS), (2) while those with different HCC trajectories had different levels of perceived insecurity (HI). We found no associations between our measures of psychosocial stress and EBV trajectories. For measures of mental health, we found no significant associations between AYMH, SDQ, CRIES symptoms and biomarker trajectories. We also found no associations between cognitive function and biomarker trajectories.

CRP trajectories were associated with levels of perceived stress. Specifically, adolescents in the Rising CRP trajectory reported fewer initial symptoms of perceived stress, relative to those in the Low CRP trajectory (*B* =  − 2.92, *SE* = 1.09, 95% CI: −5.06, −0.78, *p* = .007). Those in the Rising CRP trajectory had increasing CRP over time but – despite hypothesized links between CRP and stress – their self-reported stress levels did not rise concurrently with their increasing CRP. That is, in terms of slope, neither Rising (*B* = 0.15, *SE* = 0.22, 95% CI: −0.28, 0.58, *p* = .491) nor High (*B* = −0.17, *SE* = 0.19, 95% CI: −0.54, 0.20, *p* = .372) trajectories differed in PSS from the Low CRP trajectory.

The HCC trajectories were associated with perceived insecurity, which is a measure of levels of fear pertaining to safety and access to daily life necessities. Hypersecretion youth reported higher initial levels of insecurity than Medium Secretion youth (*B* = 7.21, *SE* = 3.02, 95% CI: 1.29, 13.14, *p* = .017); there were no differences in slope (*B* = −0.69, *SE* = 0.50, 95% CI: −1.68, 0.30, *p* = .174). That is, youth who had higher levels of cortisol secretion also reported higher initial levels of insecurity. Given that the intervention impacted HCC (see below), we re-ran these HCC analyses with treatment group as a covariate. Results were not substantively changed by this addition: as in analyses which do not control for participation in the intervention, there were differences in the intercept but not the slope of insecurity by HCC trajectories.

### 3.4 Tracking responses to the intervention

Our final research question (RQ3) tested whether participation in the 8-week psychosocial intervention predicted changes in physiology, as indexed by inflammation, immune response, or neuroendocrine stress. The intervention had a positive effect on cortisol regulation: for adolescents engaged in the intervention, relative to the control group, HCC went up at a slower rate (*B* =  − 0.10, *SE* = 0.04, 95% CI: −0.17, −0.03, *p* = .005; Table 3). That is, participants in the treatment arm of the trial showed attenuated cortisol production, reducing HCC levels by more than one-third (37.7%) across the 11-month period of study. By contrast, the intervention had no detectable impact on CRP or EBV levels (Table 3). We found no evidence for moderation by gender or trauma exposure: the intervention did not have a different effect on levels of CRP, EBV, or HCC dependent on participants’ gender or levels of trauma exposure.

**Table 3:** Three separate growth curve models showing the effects of the 8-week psychosocial intervention on C-reactive protein, Epstein-Barr virus antibodies, and hair cortisol concentration in Syrian refugee and Jordanian host-community adolescents (*n *= 817).

	*B*	*SE*	*p*	95% CI
*Outcome variables*				
C-reactive protein (CRP)	−0.01	0.03	0.869	−0.06, 0.05
Epstein-Barr Virus (EBV)	0.01	0.02	0.475	−0.02, 0.04
Hair cortisol concentration (HCC)	−0.10	0.04	0.005	−0.17, −0.03

*Note:* CRP is measured in log mg/L, EBV in log U/ml, and HCC in log pg/mg. The intervention had no detectable impact on CRP or EBV. By contrast, HCC went up at a slower rate among adolescents engaged in the intervention, relative to adolescents in the control group of the randomized controlled trial.

## 4 Discussion

This study is unique in a number of ways: it examines a gender-balanced, community-based cohort of adolescents in the context of an unfolding humanitarian crisis; describes biomarker trajectories and prospective associations with demographic characteristics, adversity, psychosocial stress, mental health, and cognitive function; and examines which biomarkers effectively track short-term responses to an intervention evaluated by means of a randomized controlled study design. Understanding the biological signatures of adversity in the wake of war and forced displacement is critical, given that they are potentially predictive of negative mental, physiological, and cognitive outcomes (Danese and McEwen, 2012, Steudte-Schmiedgen et al., 2016). Drawing on a cohort study of refugee and non-refugee adolescents, we examined the prospective trajectories of inflammation, cell-mediated immunocompetence, and neuroendocrine stress, in association with demographic characteristics and adverse experiences (RQ1), as well as outcomes related to psychosocial stress, mental health, and cognitive function (RQ2). We also evaluated biomarker responsiveness to a brief psychosocial intervention (RQ3) to mitigate young people’s experiences of profound stress. Unexpectedly, we did not observe many differences in physiological profiles between Syrian refugees and Jordanian non-refugees, nor did we find biomarker associations with exposure to lifetime trauma. We found a within-population heterogeneity of biomarker trajectories that did not necessarily map closely onto differences in adverse experiences. We also found heterogeneity in terms of which biomarker tracked changes in self-reported mental health and psychosocial stress, following a structured intervention.

In terms of our first research question, we found three distinct trajectories for markers of inflammation (high, rising, and low CRP), two for cell-mediated immunity (high and low EBV), and three for hair cortisol (HCC hyper, medium, and hyposecretion). We thus found substantial cohort heterogeneity, signaling differences in inflammatory processes, immune competence, and neuroendocrine stress across population sub-groups. These findings challenge expectations of straightforward associations between ecological context, childhood adversity, and physiology. Specifically, null or inconsistent associations with biomarker trajectories during adolescence may reflect (1) within-cohort differences in individual life history strategies, in response to levels of adversity or within-cohort differences in adverse exposures, as well as (2) latency in the time between exposure and measurable physiological changes. We discuss these possibilities below.

First, what does this study show in terms of within-cohort differences in biological responses to adversity? To elucidate what might explain the presence of distinct biological trajectories in this cohort (RQ1), we examined their associations with socio-demographic characteristics and found both expected and unexpected results. BMI has been shown to be an important confounder for CRP (Liu et al., 2017, McDade et al., 2016, Dowd et al., 2010) and HCC (Rippe et al., 2016, Stalder et al., 2017), but is unrelated to EBV (McClure et al., 2010). Indeed, we found robust associations between inflammatory response, neuroendocrine stress, and BMI. Del Giudice and Gangestad (2018) point to BMI as a marker of energy resources which is an important mediator in the physiological tradeoffs individuals need to make to maintain biological function. By contrast, there were no associations between our measured biomarkers and age. Girls, relative to boys, were more likely to have a trajectory of cortisol hypersecretion, yet they showed similar trajectories of inflammation and immunocompetence. We found no associations between biomarkers and two different measures of adversity - lifetime trauma exposure and household poverty - which might be expected to alter physiological processes, and by extension, risks to health and functioning. However, we note that, on average, Syrian participants had been re-settled in Jordan for over 2 years at the time of study, and that Jordanian participants also came from disadvantaged backgrounds, given that poor access to basic resources was a criteria for trial eligibility, which may help to explain similarities across the refugee and non-refugee sample populations.

Given the substantial differences in forced migration experiences and the burden of lifetime adversity between Syrians and Jordanians, we had expected to find distinct CRP, EBV, and HCC trajectories by refugee status. This was not the case for CRP and HCC, while results for EBV were counter-intuitive, in that Syrian refugees were more likely to have low, rather than high, EBV values. A down-regulation of immune responses may, in fact, be an adaptive response to the level and type of ecological stressors (Bonneaud et al., 2003, Segerstrom, 2010, Straub et al., 2010); perhaps Syrian refugees are facing such substantial challenges that it is not adaptive to mount a strong – and energetically costly – immune response. In absolute terms, EBV levels of study participants were comparable to those observed for US children of a similar age, for whom no associations between SES, stressful life events, and perceived stress were uncovered (Dowd et al., 2014). As Del Giudice and Gangestad (2018) remind us, “inflammatory pathways are intertwined with those that regulate stress and metabolism” (p.61), which may help to explain divergent patterns of CRP, EBV, and HCC over time. Specifically, CRP and cortisol have multiple functions: CRP is involved in both inflammatory pathways and somatic tissue repair, while cortisol production reflects both psychosocial stress adaptation and metabolic regulation. From a life history perspective, CRP levels may reflect increased investment in tissue repair and immune readiness, rather than ongoing inflammation and dysregulation, “with markedly different implications for theory and intervention” (Del Giudice & Gangestad, 2018, p. 68). As demonstrated in the Gambia, CRP levels may also reflect tradeoffs between immune function and growth during adolescence, suggesting that “investment in non-acute immune function is facultative, and sensitive to energy resources and demands” (Shattuck-Heidorn et al., 2017, p.27). Moreover, a possible confounder in such delicate tradeoffs will be gene-environmental correlation, affecting the associations between risk exposures and developmental psychopathology and the scope of environmental intervention (Jaffee and Price, 2007, Knafo and Jaffee, 2013); such a confound is plausible where both genes and behaviors potentially modify the environment, as in the case of harsh parenting, but less likely in situations where insecurity, stress and trauma arise from exposures to war, forced displacement, and poverty. In future work, it will be important to carefully measure different types of environmental adversity and reflect on what drives within-cohort differences in individual life history strategies. Our results add to a body of literature indicating that stress biomarkers are not always straightforward indicators of health-related vulnerability, as life history strategy and sensitivity to environments can influence biological variation in response to life adversity.

In our sample of war-affected adolescents, CRP levels were only mildly elevated (mean = 1.42 mg/L, median = 0.63 mg/L), ranging from 0.05 to 9.98 mg/L (excluding values > 10 mg/L, or 3% of data points, which likely indicated the presence of active infection). There were no baseline gender differences in CRP between boys and girls (mean = 1.37, median = 0.64 vs. mean = 1.47, median = 0.61 mg/L, respectively) nor between Syrian refugees and Jordanian non-refugees (mean = 1.30, median = 0.56 vs. mean = 1.56, median = 0.70 mg/L, respectively). Such levels are higher than those reported in other cohort studies, which were able to draw on population-based data. For example, median CRP levels reported in a population-based study of US youth were 0.20 mg/L for ages 9–16 and 0.75 mg/L for ages 19–21, rising with cumulative exposure to childhood bullying (Copeland et al., 2014). In a British study investigating childhood victimization, including exposure to domestic violence, bullying by peers, maltreatment, abuse, and neglect, mean CRP values were 0.65 mg/L in non-victimized 18-year olds, 0.74 mg/L for those exposed to one type of victimization, and 0.81 mg/L for those exposed to multiple events; however, the association between life adversity and CRP levels was driven by girls, for whom CRP levels were higher (respectively, 0.75 mg/L, 0.87 mg/L, 1.19 mg/L; Baldwin et al., 2018). It is clear that more work needs to be done with respect to elucidating the associations between adverse experiences and elevated CRP, especially for populations outside Western contexts, which would speak to within-cohort differences in adverse experinces and life history strategies.

Second, does this study indicate potential latency in the time between adverse exposure and measurable physiological changes? It remains unclear if life adversity – such as poverty, trauma, violence or forced displacement – produces measurable physiological changes during adolescence, without time-lag in terms of physiological dysregulation. It is possible that the impacts of risk factors on biomarkers only emerge later in life (Ben-Shlomo & Kuh, 2002). A systematic review of the effects of childhood adversity on inflammatory markers during childhood and adolescence found limited and inconsistent results (Slopen et al., 2012). Similarly, the associations between cell-mediated immune function and measures of life adversity are inconsistent: while some studies show positive associations between EBV and stressors such as discrimination and stressful life events (McClure et al., 2010, McDade et al., 2000), Dowd et al. (2014) found no associations between EBV and socioeconomic status or life events. It may be that experiences in early life, and not adolescence, predict EBV levels in young adulthood (Slopen et al., 2013b). Studies of trauma exposure have consistently associated trauma events with cortisol production in children and adolescents – albeit finding associations with both hyper- and hypocortisol secretion (Gray et al., 2018, Steudte-Schmiedgen et al., 2016). As shown in a study of war-affected Palestinian adolescents, the effects of trauma exposure on HCC are dependent on potential risk and protective factors, such as PTSD and a sense of coherence, which influence the degree to which trauma and other stressors are found manageable or meaningful (Shaheen et al., 2018). Certainly, many key developmental questions remain unanswered (Baldwin et al. 2018). We know too little about physiological plasticity during adolescence, in reponse to variation across ecological contexts. It is likely that the enduring effects of early life exposure may be due to greater physiological plasticity during early development (Gluckman et al., 2007), but too little is known about physiological plasticity during adolescence, in response to variation across ecological contexts, to anticipate the timescale of the biological effects of adverse experiences. Our results potentially suggest that adversity experienced in adolescence may be less salient than adversity experienced in early or middle childhood, and are in line with studies that indicate that evidence of physiological dysregulation may only emerge later in life (Slopen et al., 2013a, Slopen et al., 2013b).

In terms of of our second research question focusing on links to wellbeing and cognition, we examined biomarkers as indicators of vulnerability, with a view to understanding risks to long-term mental health, learning skills, and development. We found associations between a rising CRP trajectory and perceived stress, and between HCC hypersecretion and perceived insecurity, but found no links to mental health difficulties (anxiety/depression and internalizing/externalizing disorders, measured by AYMH and SDQ), posttraumatic stress (measured by CRIES), or cognitive function (measured in tablet-based experimental tests). Our finding that increasing CRP is not associated with concurrent rises in perceived stress may be explained by Del Giudice and Gangestad’s (2018) work suggesting that, in the absence of elevated levels of other inflammatory markers, moderate elevations of CRP may not reflect a pro-inflammatory state; moderate elevations of CRP may instead reflect upregulated tissue maintenance, repair in the face of chronic stress, and ongoing inflammation in response to environmental threats. A sustained activation of tissue repair mechanisms would reflect different tradeoffs in life history strategies and energy expenditure that favors somatic maintenance (Del Giudice & Gangestad, 2018).

In terms of our third research question, we found no evidence that the intervention had any impact on inflammation or immunocompetence for trial participants, relative to waitlisted controls. Given striking cortisol responses (lowering HCC by one-third), and notable reductions in self-reported symptoms of psychosocial stress and mental health, we had expected to see pre/post intervention changes for CRP and EBV. We were mindful that CRP and EBV levels are potentially more affected by short-term conditions, over timelines of 1–2 weeks, while cortisol levels in 1–2 cm of scalp hair reflect HPA activity over the timeline of 1–2 months (Russell et al., 2012). Our results suggest that different biomarkers are differentially suited to capture small-to-medium changes in adolescent wellbeing, and differentially able, in their sensitivity and specificity, to track the efficacy of behavioral or psychosocial interventions. As Slopen et al. (2014) have pointed out (p.323), we need to strengthen evidence that “adversity disrupts functioning across multiple functioning systems” – the degree to which we can “improve functioning across multiple regulatory systems is a critical question for future research.” Future work will need to evaluate the usefulness of multiple biomarkers to track the differential effects of mental health problems and psychosocial stress on various physiological systems, over short and longer timescales, in responses to interventions.

Our study has five main limitations. First, we used a limited number of biomarkers, selected carefully such that our protocol remained as field-friendly and non-invasive as possible. The collection of saliva samples, which we attempted, was found unsustainably burdensome for the population and field team; by contrast, the collection of dried blood spots by trained nurses and hair samples by professional hairdressers went smoothly. Second, samples were collected from adolescent participants at only three points, pre/post intervention and 11-month follow-up, thus we could not examine associations between adversity, biomarkers, and health outcomes over the long-term or across key developmental periods. Third, we had substantial attrition over the year of study (Dajani et al., 2018, Panter-Brick et al., 2018); in response, we used growth models robust to missing values and uneven data points (Hruschka et al., 2005, Van Ryzin et al., 2009). Fourth, potential confounders limit data interpretation; in forthcoming work, we will examine adolescent phenotypes in relation to genetic and epigenetic data collected from cheek swabs for this cohort. Finally, we may not have captured all inter-individual variation, while multiple analyses may have increased the risk of false positives.

In conclusion, it is important to document the ways in which adverse experiences are associated with inflammation, immunocompetence, and cortisol secretion, during adolescence, in both Western and non-Western contexts. This will help inform our understanding of likely health consequences and life history tradeoffs across a range of ecological contexts, and evaluate the impact of interventions targeting the period of adolescence for improved health and wellbeing. Our study is the first to examine multiple biomarker trajectories with war-affected adolescents in order to better evaluate the extent, timing, and malleability of the biological signatures of poverty, conflict, and forced displacement. Our results demonstrate the presence of distinct trajectories, signaling heterogeneity within this cohort of adolescents, and suggesting that commonly-assayed biomarkers of inflammation and immune response do not respond to targeted social and behavioral interventions in straightforward ways. Future work can further examine the etiology of gender differences, the utility of the different biomarkers which are feasible to collect in humanitarian settings, and the impact of different types of interventions for reducing risks to healthy development among war-affected youth. These questions bear on our understanding of life history and the functional significance of biomarkers in scholarly research and programmatic interventions.

## Figures and Tables

**Table A:** C-reactive protein, Epstein-Barr virus antibodies, and hair cortisol concentration values by participant gender.

	Male						Female					
	T1		T2		T3		T1		T2		T3	
	*M*	*SD*	*M*	*SD*	*M*	*SD*	*M*	*SD*	*M*	*SD*	*M*	*SD*
C-reactive protein (CRP)	−0.10	0.43	−0.15	0.38	−0.21	0.49	−0.09	0.43	−0.05	0.43	−0.20	0.45
Epstein-Barr virus (EBV)	2.00	0.38	1.97	0.35	1.95	0.39	2.07	0.36	2.07	0.37	2.04	0.40
Hair cortisol concentration (HCC)	0.73	0.32	0.52	0.59	0.82	0.56	0.90	0.39	0.79	0.56	1.05	0.50

*Note:* CRP is measured in log mg/L, EBV in log U/ml, and HCC in log pg/mg. At T1, there were 465 male and 352 female participants.
