# Supplementary material for: C-reactive protein, Epstein-Barr virus, and cortisol trajectories in refugee and non-refugee youth: Links with stress, mental health, and cognitive function during a randomized controlled trial
Source: Brain Behav Immun. 2020 Jul;87:207–17. doi: 10.1016/j.bbi.2019.02.015 (PMC7327518; doi:10.1016/j.bbi.2019.02.015)
Supplement: Supplementary data 1 [file mmc1.docx]

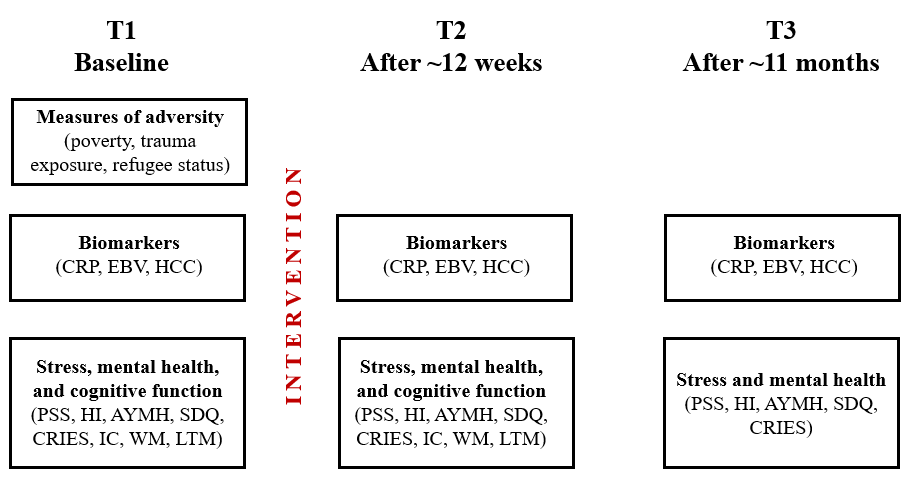


*Supplemental Figure A.* Data collection timeline.

Note*:* CRP = C-reactive protein, EBV = Epstein-Barr virus, AYMH = Arab Youth Mental Health scale, SDQ = Strengths and Difficulties Questionnaire, CRIES = Child Revised Impact of Events Scale, PSS = perceived stress scale, HI = Human Insecurity scale, IC = inhibitory control, WM = working memory, LTM = long-term memory.
